# Three-dimensional nephrometry scoring system: a precise scoring system to evaluate complexity of renal tumors suitable for partial nephrectomy

**DOI:** 10.7717/peerj.8637

**Published:** 2020-02-27

**Authors:** Jingchao Liu, Jing Liu, Shuo Wang, Haifeng Zhao, Chuanxin Tian, Benkang Shi, Xianzhou Jiang

**Affiliations:** 1 Department of Urology, Qilu Hospital of Shandong University, Jinan, Shandong Province, China; 2 Department of Biostatistics, School of Public Health, Shandong University, Jinan, Shandong Province, China

**Keywords:** Kidney neoplasms, Nephrectomy, Three-dimensional imaging, Partial nephrectomy, Warm ischemic time

## Abstract

**Purpose:**

Several nephrometry scoring systems have been developed based on two-dimensional computerized tomography images to quantify anatomical features of renal tumors. We have developed an accurate three-dimensional nephrometry scoring system to respond to the urgent need for advanced systems based on three-dimensional images.

**Materials and Methods:**

We retrospectively reviewed 135 patients who underwent partial nephrectomy in our institution. Stereoscopic models were reconstructed from preoperative computerized tomography images and three-dimensional scores were assigned directly on stereoscopic models. All tumors were analyzed for following features: tumor volume; endophytic tumor proportion; renal vascular variations; tumor’s relationships with urinary collecting system or renal sinus; longitudinal distance from tumor to equatorial plane. Correlation between three-dimensional score and warm ischemic time was calculated compared with existing classical nephrometry scoring systems. The value of nephrometry scoring systems predicting longer warm ischemic time was explored by receiver operating characteristic curves.

**Results:**

Mean tumor volume was 31.25 ml; endophytic volume was less than 50% in 42 cases, more than 50% in 79 cases, and 100% in 14 cases; mean longitudinal distance from tumor to equatorial plane was 1.41 cm; 30 patients (22.2%) presented renal vascular variations; 18 cases (13.3%) involved both urinary collecting system and sinus. Mean three-dimensional score was 8.3. Variance analysis and covariance analysis revealed warm ischemic time a significant association with all evaluated tumor features. Furthermore, three-dimensional scores most highly correlated with warm ischemic time (rs = 0.64, *p* < 0.001), followed by R.E.N.A.L. scores (rs = 0.21, *p* = 0.012), centrality index (rs = − 0.20, *p* = 0.019) and Preoperative Aspects and Dimensions Used for Anatomy score (rs = 0.20, *p* = 0.019). Area under curve of above nephrometry scoring systems was 0.91, 0.67, 0.68 and 0.67 respectively (*p* < 0.05).

**Conclusions:**

The three-dimensional scoring system developed in this study was a highly-accurate system to quantify the anatomical features of renal tumors. It was identified to have a value in predicting duration of warm ischemic time.

## Introduction

Partial Nephrectomy (PN) surgery was recommended as the gold standard of care for tumors ≤4 cm (T1a) by American Urological Association and European Association of Urology guidelines (Campbell et al., 2009; Ljungberg et al., 2015). It was also suggested for tumors of 4–7 cm (T1b) because of the therapeutic equivalency compared with radical nephrectomy (Turna, Aron & Gill, 2008).

**Figure 1:**
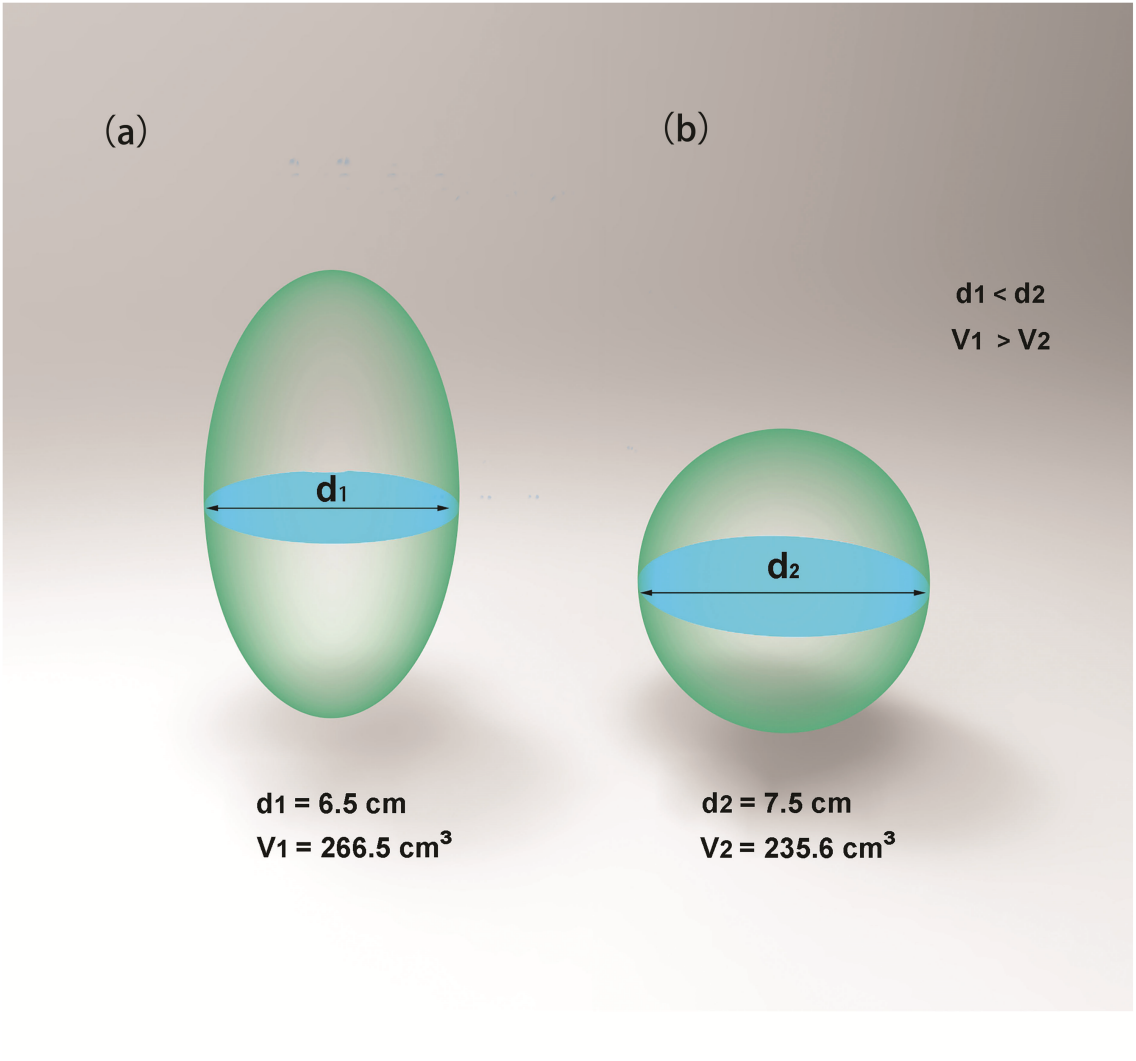
Tumors with a longer diameter in 2D cross-sections may hold a smaller volume in 3D images. Although diameter of ellipsoid 1 (A) is smaller than diameter of ellipsoid 2 (B) (d1 < d2), volume of ellipsoid 1 is larger than that of ellipsoid 2 (v1 > v2).

Clinicians and radiologists had developed several nephrometry scoring systems to quantify anatomical features of renal cell carcinoma. The most popular systems such as R.E.N.A.L. (radius, exophytic/endophytic properties, nearness of tumor to collecting system or sinus, anterior/posterior, location relative to polar lines and tumor touching main renal artery or vein) score, PADUA (preoperative aspects and dimensions used for anatomy) score and CI (centrality index) score could help surgeons to objectively predict the complexity of renal tumors and various perioperative outcomes (Kutikov & Uzzo, 2009; Ficarra et al., 2009; Simmons et al., 2010).  These nephrometry scoring systems greatly improved surgical planning of clinicians and counseling of patients. However, existing nephrometry scoring systems were associated with various limitations (Kolla, Spiess & Sexton, 2011; Okhunov et al., 2011; Monn et al., 2014; Benadiba et al., 2015).  Each score’s predictive ability was inconsistent between different researches and score-reader’s experience also affected reproducibility of nephrometry scoring systems. Meanwhile, renal tumor volume was found to have more predictive value for renal function outcome compared to traditional tumor diameter (Mir et al., 2013; Liss et al., 2016). Existing nephrometry scoring systems were assigned mainly by analyzing traditional two-dimensional CT images. In nature, two-dimension (2D) has its limitations and inaccuracy compared with three-dimension (3D): (1) Tumors with a longer diameter in 2D cross-sections may hold a smaller volume in 3D images, which is illustrated intuitively by Fig. 1. (2) 2D images cannot precisely present the condition of renal vascular variation, while this can be illustrated in 3D images. (3) 3D images can provide more accurate imagery of many anatomical features. We can rotate 3D models at different angles to achieve the optimal effect. We developed a 3D scoring system which could improve radiological evaluation of renal tumors. This 3D scoring system also had a predictive value for duration of warm ischemic time (WIT) in our study.

## Materials and Methods

We reviewed 135 consecutive patients who underwent laparoscopic PN for renal tumors between November 2016 and January 2018. All patients gave written informed consents for publication. Our case study was approved by Qilu Hospital Review Board (Approval number: 2017067) and was accordant with Helsinki II declaration. All cases met our study inclusion criteria of a single tumor excision, a contralateral symmetrical kidney, no allergies to enhanced contrast agent, available preoperative enhanced CT images and postoperative pathological findings of renal clear cell carcinoma. All operations were performed extraperitoneally by one experienced surgeon (Professor Jiang), having performed 500 + PNs before. Tumors were identified using intraoperative ultrasound. After dissection of perirenal fat from kidney, the tumor was exposed. Renal artery was clamped with bulldogs when necessary. A safety margin, approximately five mm larger than normal tumor boundary, was guaranteed during tumor excision. Repair of collecting system and reconstruction of the parenchymal defect was completed with techniques well described previously (Porpiglia et al., 2008; Mottrie, Borghesi & Ficarra, 2012). WIT was accurately recorded during operation. All cases underwent contrast-enhanced CT scan preoperatively with multi-slice CT system (SOMATOM Force, Siemens, Erlangen, Germany). The scanning protocol was as follows: rotation time: 0.5 s, 100 kv; slice thickness: 1.0 mm; contrast medium: Ultravist (300 mg I/ml) at a rate of 4.5 m1/s. The enhanced images (including arterial, venous and excretory phases) were then input into 3D rendering software (CSA, Hisense, Tsingtao, China) for post-processing. CSA could efficiently render tumor areas and endophytic tumor areas, then built up visualized 3D models. Basing on the 3D models, software could automatically calculate tumor volume, endophytic tumor volume and longitudinal distance from tumor to equatorial plane (LDTE). It can also demonstrate the whole anatomical structures from different angles. The 3D score measurements were made directly on 3D models.

**Figure 2:**
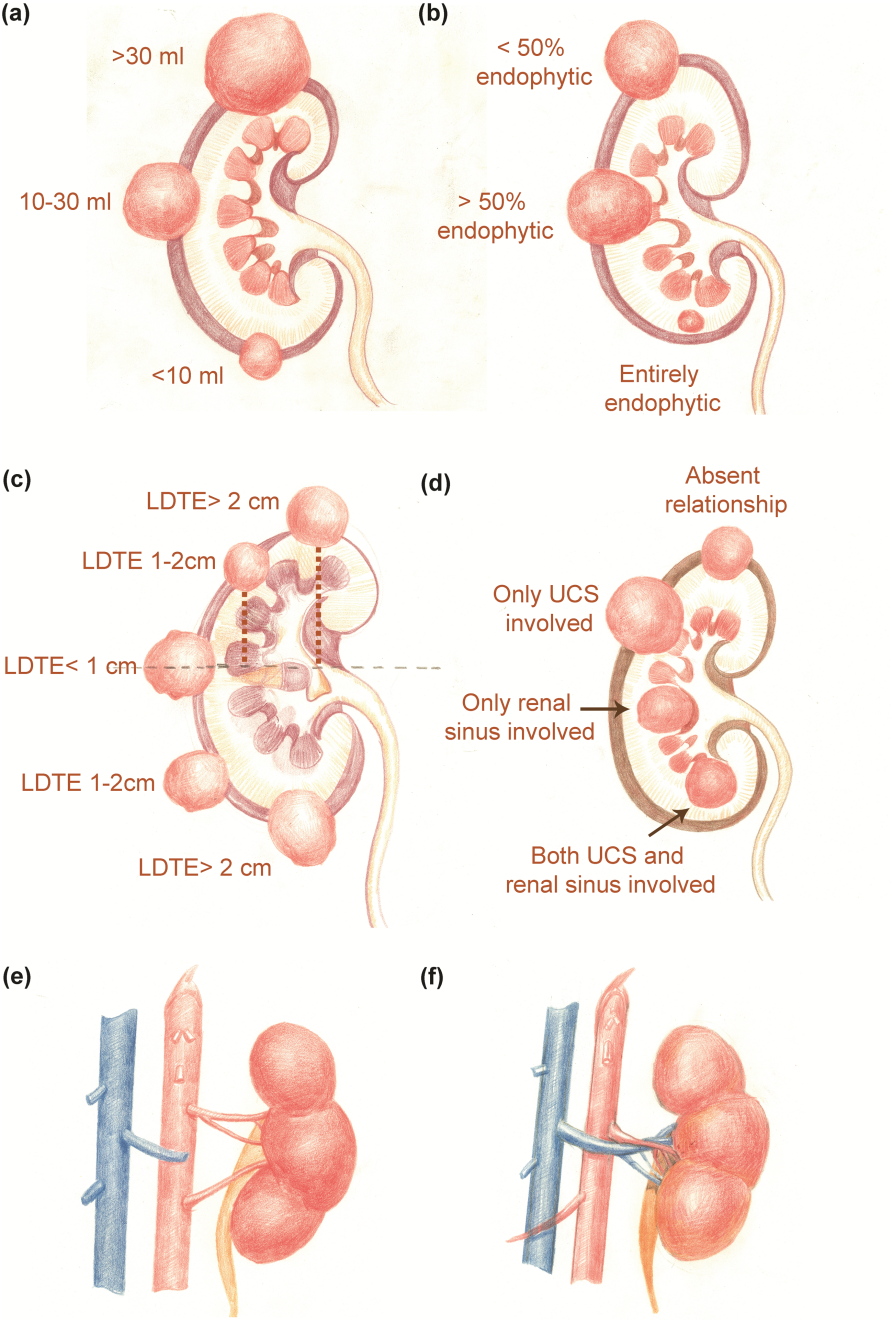
3D nephrometry score elements. (A) Tumor volume classification. (B) Endophytic/exophytic proportion classification. (C) Longitudinal distance classification. (D) Relationship with UCS and renal sinus. (E) Renal vascular variation for number of feeding arteries. (F) Renal artery running anteriorly to renal vein.

**Figure 3:**
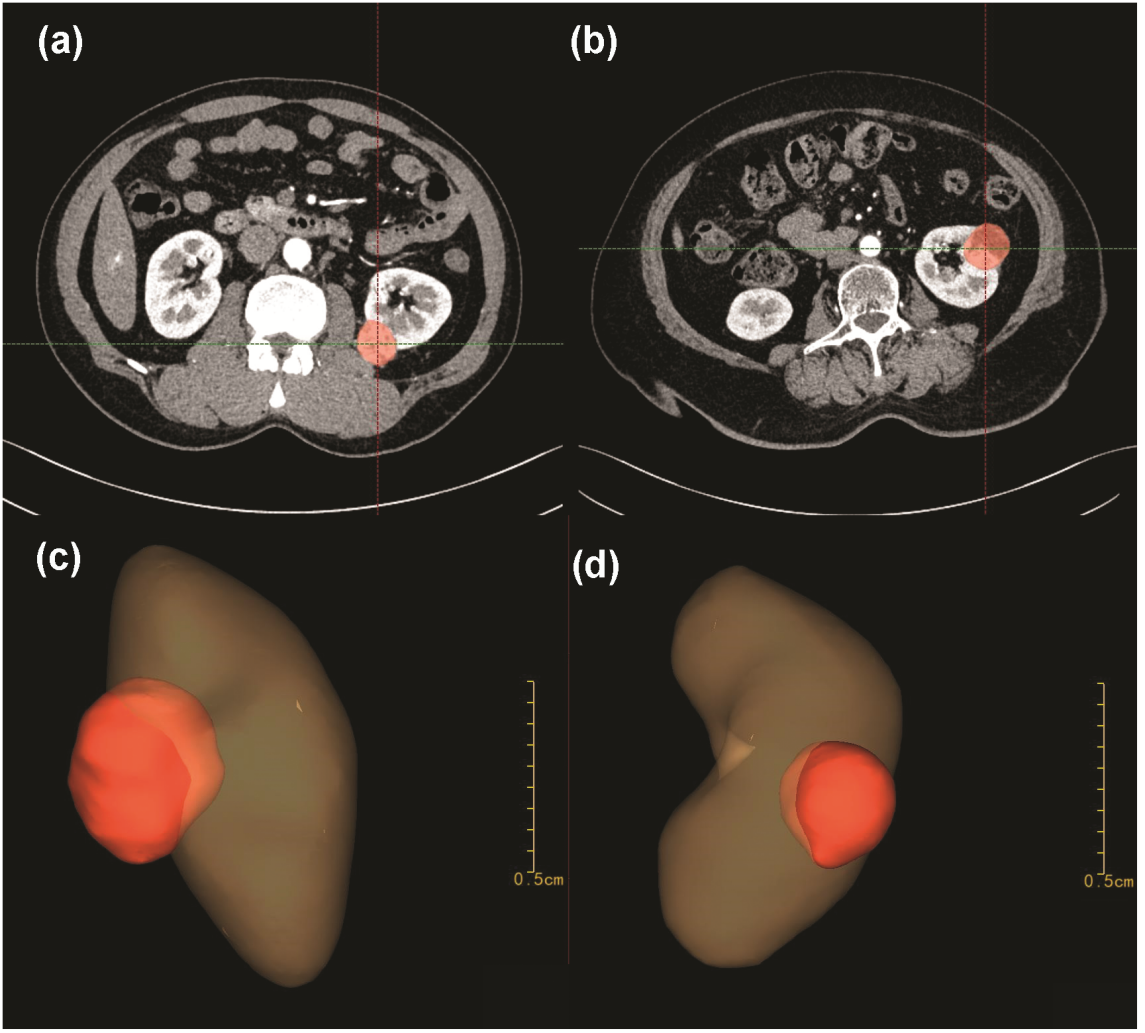
A longer 2D diameter was not equal to a larger 3D volume in our cases. Patient A measured a shorter diameter (A) in 2D CT images than patient B (B). After 3D reconstruction, patient A (C) measured a greater volume than patient B (D).

**Figure 4:**
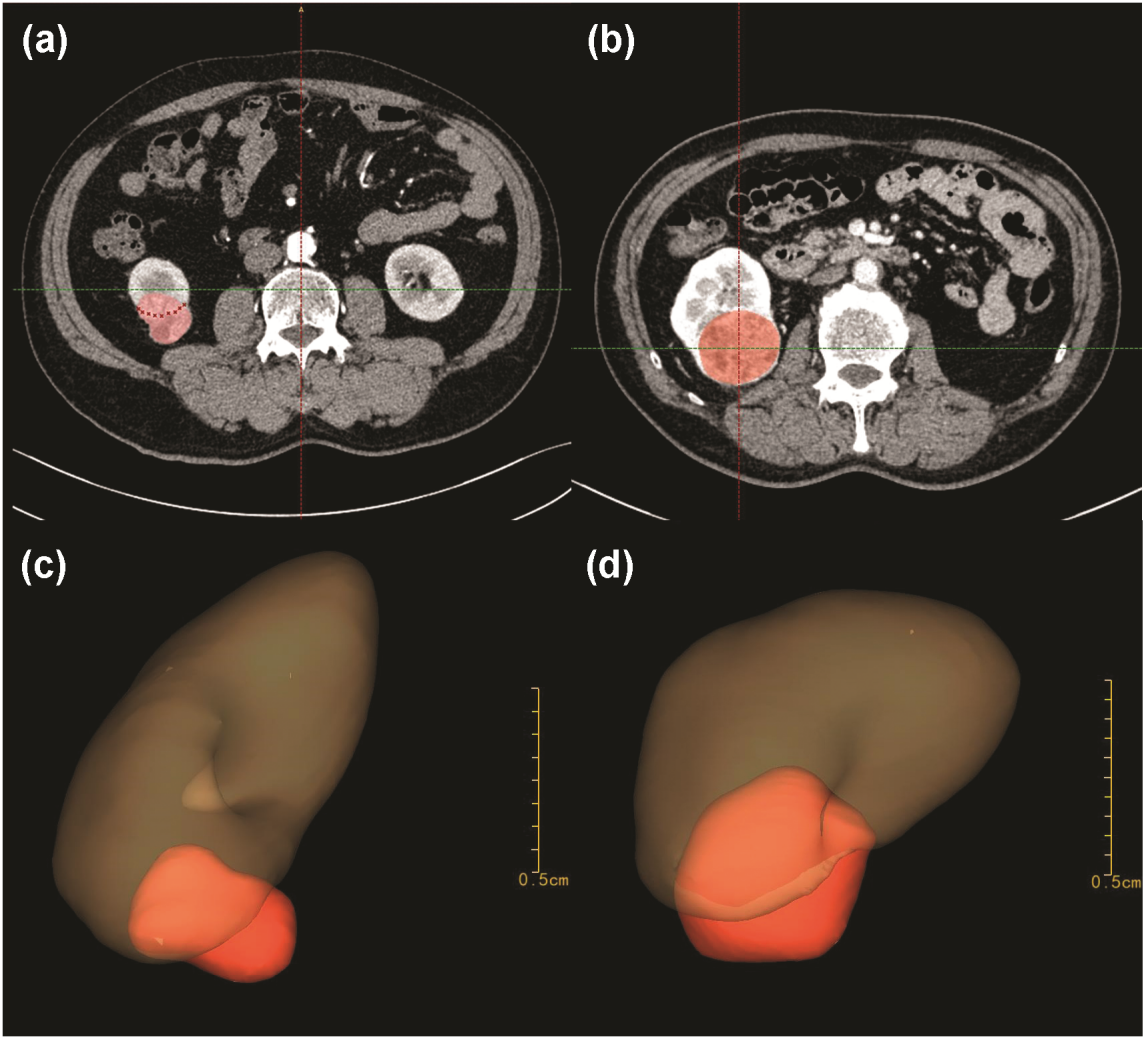
Inconsistency of endophytic-percentage between 2D and 3D dimensions. Patient C in Fig. 4 measured a <50% endophytic characteristic in traditional 2D CT images (A). But the patient measured a >50% endophytic characteristic in 3D models (C). Patient D in Fig. 4 presented an entirely-endophytic characteristic in 2D cross-section, which section showed the tumor’s longest diameter (B). After 3D reconstruction, the same patient presented a >50% endophytic characteristic in 3D models (Fig. 4D).

Tertiles division was used to describe the distribution of the whole volume data: (1) less than 10 ml; (2) 10–30 ml and (3) greater than 30 ml (Fig. 2A). Endophytic/exophytic volume rate also was classified into three groups: (1) <50% endophytic; (2) ≥50% endophytic and (3) entirely endophytic (Fig. 2B). A longer diameter in 2D dimension was not equal to a larger volume in 3D dimension (Fig. 3). Figure 4 also demonstrated the inconsistency in endophytic/exophytic ratios between 3D images and 2D images. According to LDTE, tumors were classified into three groups: (1) Less than one cm; (2) 1–2 cm and (3) greater than two cm (Fig. 2C). With respect to UCS or renal sinus, tumors were also categorized into three groups: (1) Absence of any involvement with UCS and renal sinus; (2) Involving either UCS or renal sinus and (3) simultaneous involvement of UCS and renal sinus (Fig. 2D).

Normally, each kidney is fed by only one renal artery. Renal veins usually run in front of the renal artery. Various kinds of renal vascular variations have been reported, which include accessory renal arteries, prehilar branching arteries, multiple renal veins as well as arteries or veins from unusual origins. Considering renal artery clamp-procedure during PN performance, only the following three kinds of vascular variants were included in our scoring system: a. Accessory renal arteries; b. Prehilar branching arteries; c. Renal arteries anterior to the veins. These variations were illustrated in Fig. 2E and Fig. 2F. If there were such variations as accessory renal arteries or prehilar branching arteries, the intraoperative-clamp of only one artery might lead to hemorrhage and worse surgical field. Furthermore, surgeons might clamp renal veins by mistake if renal arteries were anterior to the veins. Tumors were consequently categorized into two groups regarding above three variations: (1) No vascular variations; (2) Variations of renal vessels.

Every category was scored on 1, 2 or 3 point scale, which were added up to get our 3D score. 3D scoring system was evaluated separately by one radiologist and two urologists, who were blinded to all patients’ outcomes. The average score was used in our cohort. R.E.N.A.L. score, PADUA score and CI score were also measured based on traditional 2D cross-sectional CT images. The tumor diameter meant the longest diameter in any 2D cross-sections. The 2D endophytic/exophytic properties were measured either in CT cross-sections which presented the longest tumor diameter or in 2D coronal planes, when necessary.

We analyzed all data using SAS v.9.4 software (SAS Institute Inc., Cary, NC, USA). Normally distributed variables were presented as the mean ± SD and nonparametric continuous data as median and interquartile range (IQR). Student *t* test, Pearson *x*^2^ and Mann–Whitney *U* test were performed as appropriate. Variance analysis and covariance analysis were used to assess relationships between 3D components and WIT. Correlation between nephrometry scoring systems and WIT was also calculated using Spearman correlation coefficient. The value of nephrometry scoring systems predicting longer WIT was also explored by receiver operating characteristic (ROC) curves. A two-sided *p* < 0.05 was viewed statistically significant.

## Results

Table 1 showed baseline characteristics in our cohort. The mean tumor diameter was 2.53 (IQR: 1.70–3.00) cm, mean tumor volume was 31.25 (IQR: 6.07–28.59) ml, and median LDTE was 1.41 (IQR: 0.00–2.12) cm. The tumor volume was <10 ml in 55 patients (40.7%), 10-30 ml in 50 patients (37.1%) and >30 ml in 30 patients (22.2%). Tumor volume was <50% endophytic in 42 patients (31.1%), >50% endophytic in 79 patients (58.5%), and entirely endophytic in 14 patients (10.4%). The LDTE was >2 cm in 41 patients (30.4%), 1–2 cm in 50 patients (37.0%) and <1 cm in 44 patients (32.6%). In 92 cases (68.2%), neither UCS nor renal sinus was involved, in 25 patients (18.5%) one of the two structures was involved and in 18 cases (13.3%) both two structures were involved at the same time. Renal vascular variation was identified in 30 patients (22.2%). The median 3D score was 8.3 (IQR: 7–10), median R.E.N.A.L. score 7.01 (IQR: 6–8), median PADUA score 8.19 (IQR: 7–9) and median CI score 3.12 (IQR: 1.97–3.73) Table 1.

**Figure 5:**
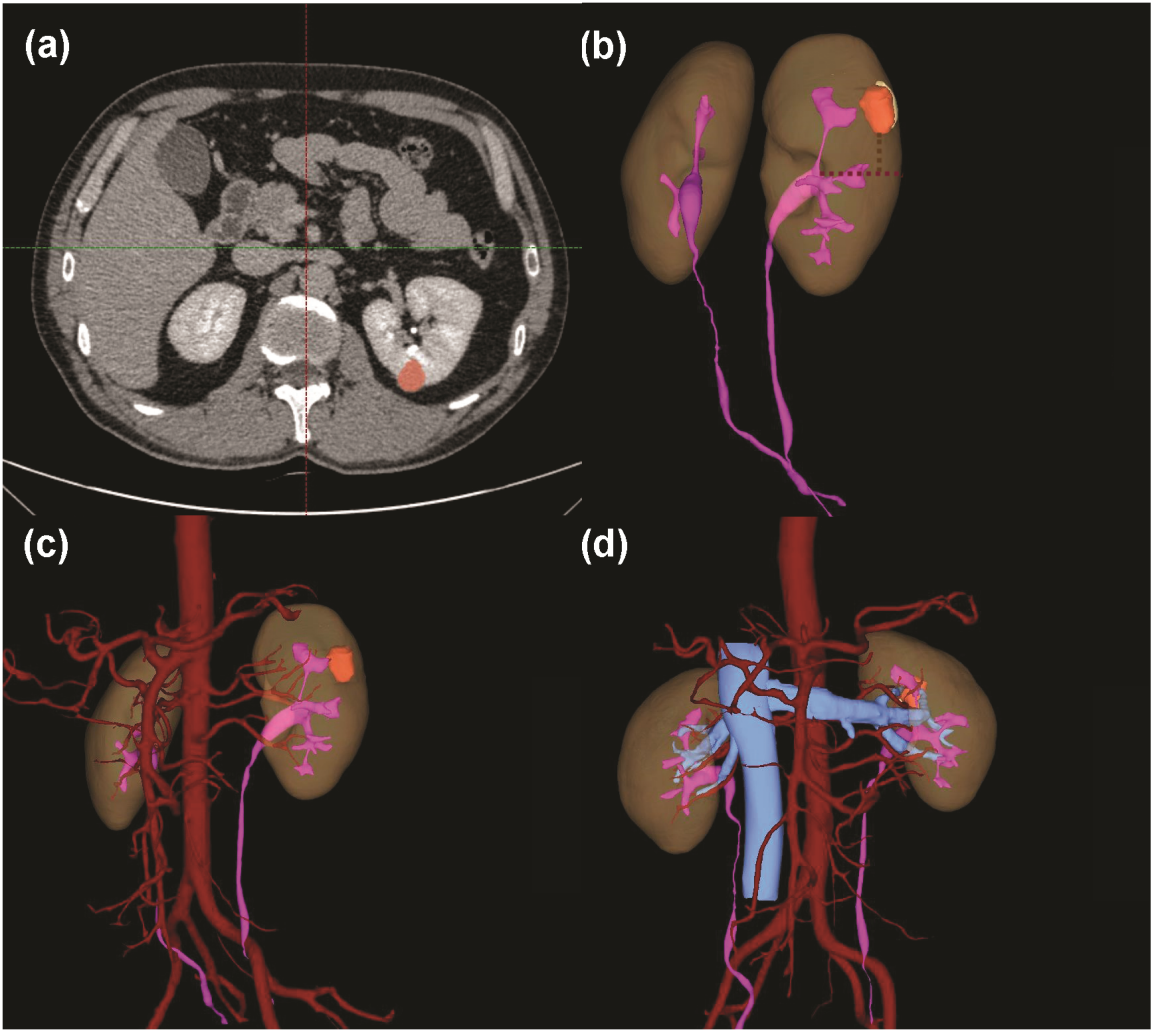
A case with a 3D score of 8. (A) Tumor identification in 2D cross-section. (B) Total tumor volume: 2.05 ml; endophytic/exophytic volume rate: >50% endophytic; LDTE: 1.78 cm; tumor’s relationships with UCS or renal sinus: neither UCS nor sinus was involved; (C) Renal vascular variation: three feeding arteries of left kidney. (D) Relative position variation of renal artery and renal vein: no variation.

**Figure 6:**
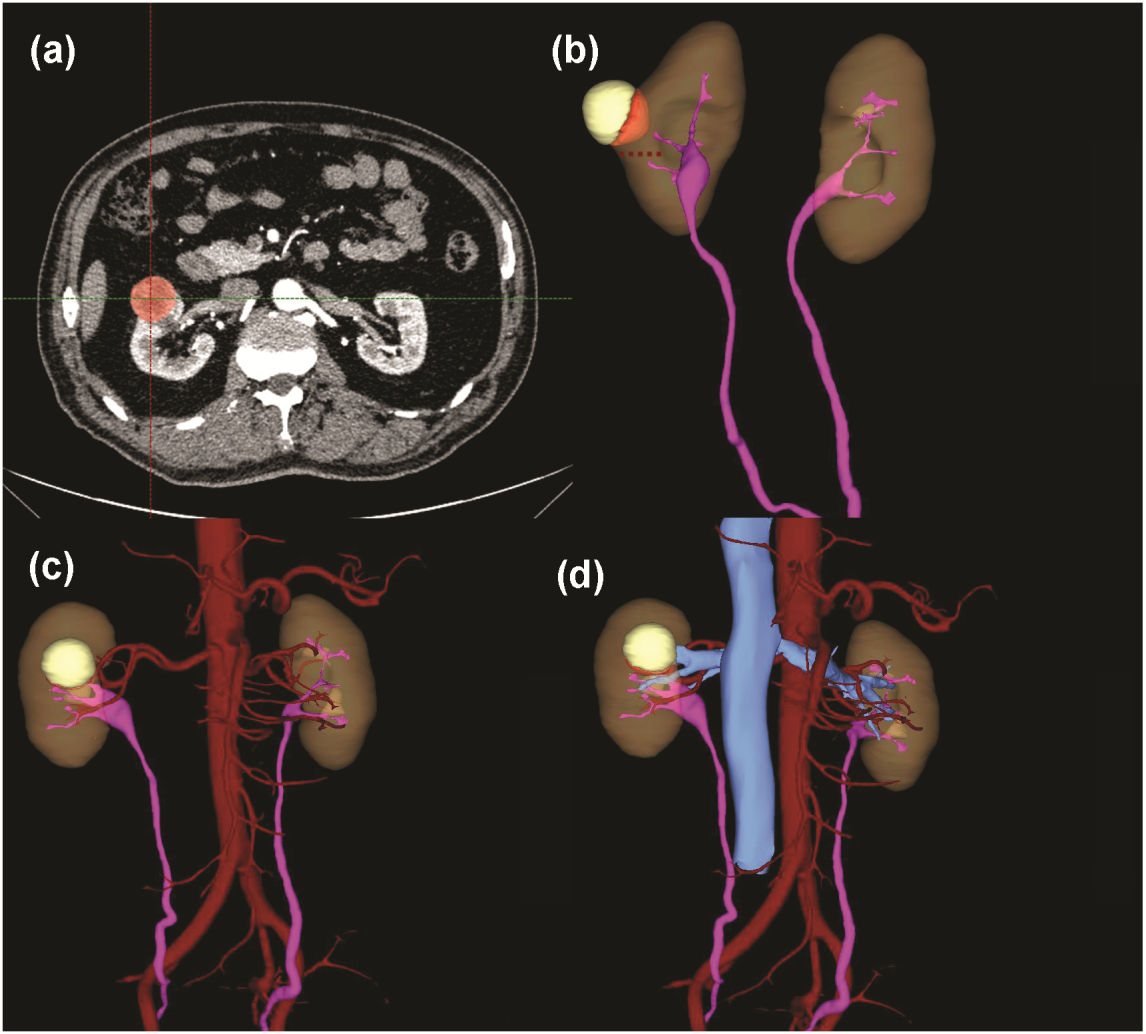
A case with a 3D score of 8. (A) Tumor identification in 2D cross-section. (B) Total tumor volume: 17.93 ml; endophytic/exophytic volume rate: <50% endophytic; LDTE: 0.88 cm; tumor’s relationships with UCS or renal sinus: neither UCS nor sinus was involved. (C) Variations of renal artery: no variation. (D) Relative position variation of renal artery and renal vein: no variation.

**Figure 7:**
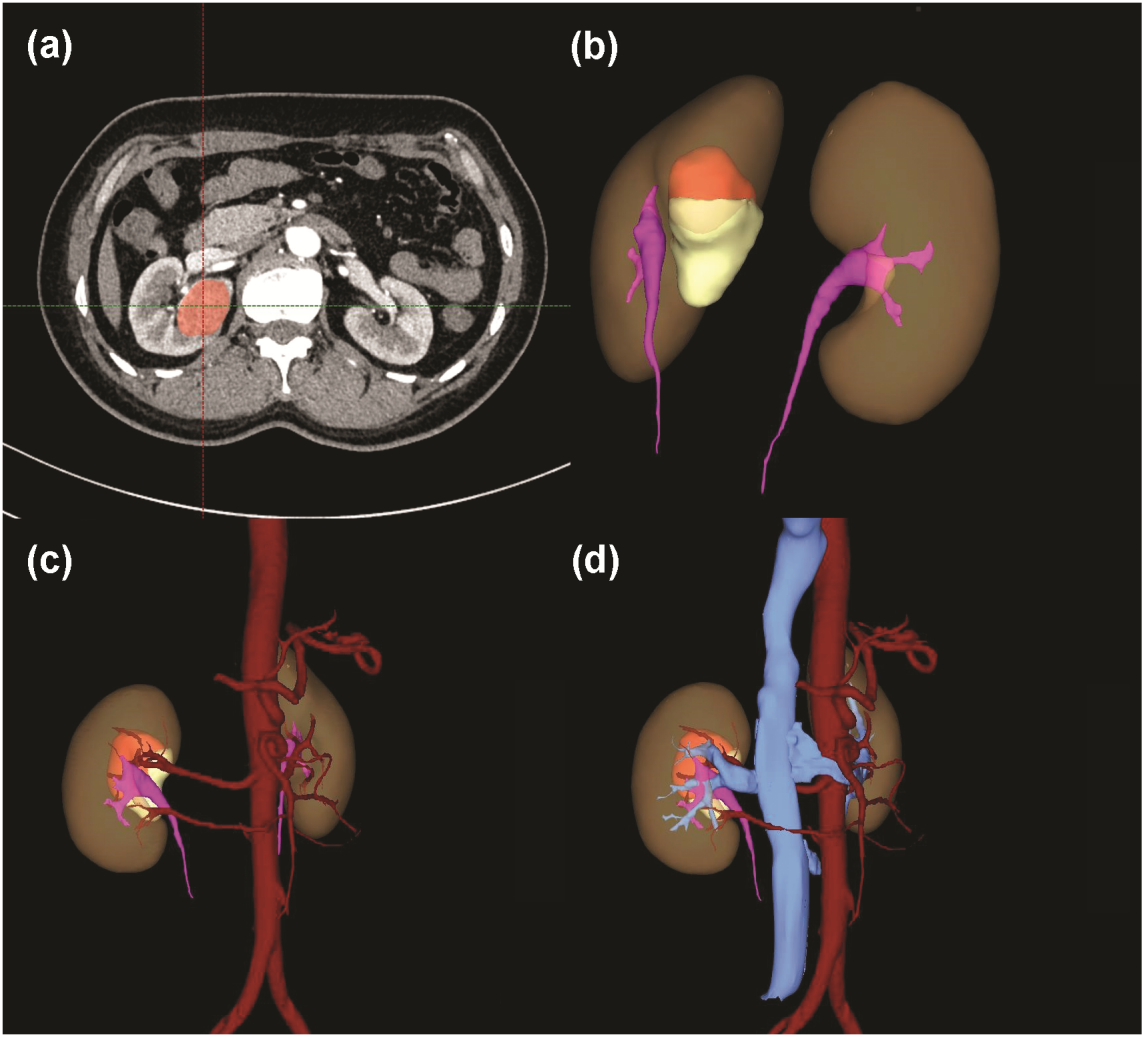
A case with a 3D score of 12. (A) Tumor identification in 2D cross-section. (B) Total tumor volume: 31.00 ml; endophytic/exophytic volume rate: <50% endophytic; LDTE: 0 cm; tumor’s relationships with UCS or renal sinus: sinus was involved. This tumor did not involve UCS, but UCS may be damaged in tumor excision process because of the close distance, so a score of 3 was signed. (C) Variations of renal artery: two feeding arteries of right kidney. (D) Relative position variation of renal artery and renal vein: no variation.

Table 2 summarized 3D score methodology in detail. Figure 1 theoretically illustrated that a longer two-dimensional diameter was not equal to a larger 3D volume. Figures 3 and 4 illustrated the same point in our practical applications. Figures 5–7 showed three representative cases of our 135 patients. Figure 5 presented a case with a 3D score of 8. Left kidney had three feeding arteries (Fig. 5C), which added great complexity while performing PN. After concealing kidney arteries and veins, and rotating to appropriate angle, we observed that tumor did not involve either UCS or renal sinus. Figure 6 also presented a case with a 3D score of 8. Figure 7 presented a case with a 3D score of 12. The large tumor invaded kidney equatorial plane and extended to hilar center point. Accessory renal artery was found for right kidney (Fig. 7C). This tumor did not involve UCS, but UCS may have been damaged during the tumor excision process because of the small distance (Fig. 7B). All above 3D features contributed to a 3D score of 12, which indicated a high complexity score in our cohort.

In variance analysis without adjusting other factors by SAS, WIT was significantly associated with tumor volume score (*p* = 0.004), endophytic/exophytic volume rate score (*p* = 0.014), LDTE score (*p* = 0.010), score of relationship with UCS or renal sinus (*p* = 0.026) and vascular variation score (*p* = 0.006). When adjusting genders, ages, lateral location and tumor face, WIT was significantly associated with tumor volume score (*p* = 0.005), endophytic/exophytic volume rate score (*p* = 0.018), LDTE score (*p* = 0.006), score of relationship with UCS or renal sinus (*p* = 0.032) and vascular variation score (*p* = 0.007) Table 3

**Table 1:** Patient and tumor characteristics.

	Overall
No. pts	135
No. male (%)	90 (66.7%)
Median age (IQR)	55 (47–62)
No. left kidney (%)	62 (45.9%)
No. anterior face (%)	77 (57.0%)
Median cm tumor largest diameter (IQR)	2.5 (1.7–3.0)
Median ml tumor volume (IQR)	31.3 (6.1–28.6)
Median cm LDTE (IQR)	1.4 (0.0–2.1)
Median R.E.N.A.L. score (IQR)	7.0 (6–8)
Median PADUA score (IQR)	8.2 (7–9)
Median centrality index score (IQR)	3.1 (2.0–3.7)
Median 3D score (IQR)	8.3 (7–10)
Median mins WIT (IQR)	28 (27–30)
No. occurrence of vascular variation (%)	30 (22.2%)
No. tumor volume (%)	–
<10 ml	55 (40.7%)
10–30 ml	50 (37.1%)
>30 ml	30 (22.2%)
No. 2D endophytic/exophytic area rate	
<50% endophytic	47 (34.8%)
50%–100% endophytic	67 (49.6%)
Entirely endophytic	21 (15.6%)
No. 3D endophytic/exophytic volume rate	
<50% endophytic	42 (31.1%)
50%–100% endophytic	79 (58.5%)
Entirely endophytic	14 (10.4%)
No. LDTE (%)	
>2 cm	41 (30.4%)
1–2 cm	50 (37.0%)
<1 cm	44 (32.6%)
No. UCS or renal sinus involvement (%)	
None-involved	92 (68.2%)
Only one parameter involved	25 (18.5%)
Both involved	18 (13.3%)
No. 3D score	
5–7	57 (42.2%)
8–10	53 (39.3%)
11–14	25 (18.5%)

**Notes.**

Abbreviations LDTE longitudinal distance from tumor to equatorial plane WIT warm ischemic time UCS urinary collecting system 2D two-dimension 3D three-dimension

**Figure 8:**
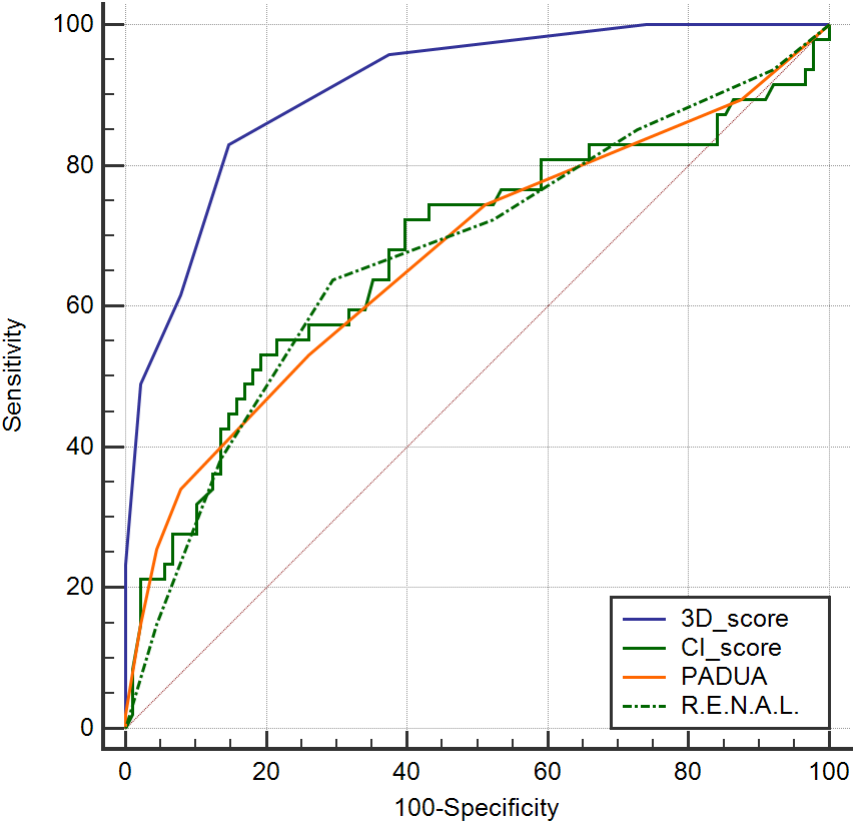
ROC curves for 3D scores, R.E.N.A.L. scores, PADUA scores and CI scores predicting WIT > 30 min.

Table 4 presented the analysis of Spearman correlation between the nephrometry scoring systems and WIT. The 3D scoring system showed strongest correlation with WIT (rs = 0.64, *p* <0.001), followed by R.E.N.A.L. scores (rs = 0.21, *p* = 0.012), centrality index (rs = −0.20, *p* = 0.019) and Preoperative Aspects and Dimensions Used for Anatomy score (rs = 0.20, *p* = 0.019). Previous studies had pointed that renal function after PN could recover efficiently as long as WIT was kept below 30 min (Becker et al., 2009; Mir et al., 2015). So WIT lasting longer than 30 min in our study was viewed as a harmful outcome. The predictive value of nephrometry scoring systems for longer WIT (>30 mins ) was explored by ROC curve analysis. Area under ROC curve (AUC) of 3D scores, R.E.N.A.L. scores, centrality index scores and PADUA scores was 0.91, 0.67, 0.68 and 0.67 respectively (*p* < 0.05). (Table 5 and Fig. 8) Analysis in the study indicated that 3D scoring system could be helpful in evaluating tumor complexity and also in predicting the duration of WIT.

**Table 2:** Score assigned to each anatomical feature included in 3D scoring system.

Anatomical features	Score
Tumor volume (ml)	
<10	1
10–30	2
>30	3
Endophytic/exophytic volume rate	
<50% endophytic	1
≥50% endophytic	2
Entirely endophytic	3
Occurrence of vascular variation	
No	1
Yes	2
LDTE	
>2 cm	1
1–2 cm	2
<1 cm	3
UCS or renal sinus involvement	
None-involved	1
Only one parameter involved	2
Both involved	3

**Notes.**

Abbreviations LDTE longitudinal distance from tumor to equatorial plane UCS urinary collecting system 3D three-dimension

## Discussion

There has been an increase in the incidence of small-sized renal tumors by CT scanning or ultrasonography (Janzen et al., 2003). PN was proposed as golden management of small-sized renal tumor (T1a) (Campbell,2009). The performance of PN depended largely on anatomical complexity of renal tumors and experienced skills of surgeons. During past decades, various nephrometry scoring systems had been developed to objectively quantify anatomical features for renal tumors (Kutikov & Uzzo, 2009; Ficarra et al., 2009; Simmons et al., 2010; Hakky et al., 2014; Nisen et al., 2014; Tannus, Goldman & Andreoni, 2014; Tobert et al., 2015; Zhou et al., 2015; Spaliviero et al., 2016). The most widely used nephrometry scoring systems included R.E.N.A.L., PADUA, and centrality index. R.E.N.A.L. and PADUA had extremely similar parameters based on 2D CT scans: the size of tumor, exophytic/endophytic properties, relationship with UCS or renal sinus and longitudinal locations. The distance from tumor center to renal center was divided by tumor radius to get centrality index score, which provided useful quantification of tumor centrality.

Many studies proved previous nephrometry scoring systems as quantitative and reproducible tools to describe the anatomical complexity of tumors (Kopp et al., 2014). However, with the in-depth research, limitations and inconsistencies of previous nephrometry scoring systems have also been demonstrated in many studies (Kolla, Spiess & Sexton, 2011; Okhunov et al., 2011; Hew et al., 2011). To make up the insufficiency of previous nephrometry scoring systems, several second-generation nephrometry scoring systems were developed in the recent years (Nisen et al., 2014; Tannus, Goldman & Andreoni, 2014; Tobert et al., 2015; Zhou et al., 2015; Spaliviero et al., 2016). Those second-generation nephrometry scoring systems needed further external validation to become popular in clinic. We proposed a 3D scoring system which involved description of relevant anatomical structures from 3D images to improve accuracy of nephrometry scoring systems.

**Table 3:** Variance and covariance analysis for warm ischemic time and 3D score’s elements.

	Model 1	Model 2^a^	Model 3^b^
	Mean ± SE	Mean ± SE	Mean ± SE
Tumor volume			
1	27.51 ± 0.69	27.51 ± 0.56	27.48 ± 0.57
2	29.36 ± 0.58	29.38 ± 0.59	29.42 ± 0.60
3	30.53 ± 0.20	30.51 ± 0.77	30.48 ± 0.77
*P*	0.004	0.005	0.005
Endo/exophytic rate			
1	27.67 ± 0.63	27.67 ± 0.65	27.66 ± 0.66
2	29.05 ± 0.49	29.06 ± 0.48	29.07 ± 0.48
3	31.43 ± 0.93	31.36 ± 1.13	31.32 ± 1.14
*P*	0.014	0.017	0.018
Vascular variation			
1	28.32 ± 0.40	28.33 ± 0.41	28.31 ± 0.42
2	30.77 ± 0.83	30.75 ± 0.77	30.80 ± 0.79
*P*	0.006	0.006	0.007
LDTE			
1	27.66 ± 0.38	27.64 ± 0.66	27.58 ± 0.67
2	28.52 ± 0.53	28.51 ± 0.59	28.43 ± 0.60
3	30.39 ± 0.86	30.41 ± 0.63	30.56 ± 0.65
*P*	0.010	0.009	0.006
UCS/sinus involvement			
1	28.23 ± 0.44	28.24 ± 0.44	28.25 ± 0.44
2	29.72 ± 0.52	29.67 ± 0.85	29.64 ± 0.86
3	30.94 ± 1.38	30.97 ± 1.00	30.96 ± 1.01
*P*	0.026	0.028	0.032

**Notes.**

a Adjusted for age and gender.

b Adjusted for age, gender, lateral location and locational face.

Abbreviations LDTE longitudinal distance from tumor to equatorial plane UCS urinary collecting system 3D three-dimension

**Table 4:** Spearman correlation analysis for nephrometry scoring systems and warm ischemic time.

	Spearman’s rs	*p*
3D score	0.64	<0.001
PADUA	0.20	0.025
CI score	−0.202	0.019
R.E.N.A.L.	0.216	0.012

**Notes.**

Abbreviations 3D three-dimension CI centrality index

**Table 5:** Analysis of different nephrometry scoring systems predicting WIT >30 min in ROC curve.

	AUC	95% CI	Sensitivity	Specificity	Cut-off
3D score	0.91	0.86–0.96	0.83	0.85	8.5
PADUA	0.67	0.57–0.77	0.53	0.74	8.5
CI score	0.68	0.59–0.75	0.53	0.81	2.2
R.E.N.A.L.	0.67	0.58–0.77	0.64	0.70	7.5

**Notes.**

Abbreviations 3D three-dimension CI centrality index WIT warm ischemic time ROC receiver operating curves AUC area under the ROC curve

Tumor size played a vital role in PN performance. Previous studies quantified tumor size with 2D tumor diameter, which meant the longest diameter measured in any 2D cross-sections. Tumor diameter did not reflect actual size of tumor to some extent (Figs. 1, 3 and 4). Only 3D volume can reflect actual size of renal tumor. Additionally, several researchers concluded that it was the 3D volume, not the two-dimensional diameter that needed to be measured to determine the functional outcomes after PN (Mir et al., 2013). Though volume measurement depending on 3D reconstruction was time-consuming compared with two-dimensional diameter, we thought it deserved our efforts because it greatly improved accuracy.

Exophytic/endophytic properties were also included in 3D scoring systems. R.E.N.A.L. suggested that axial, coronal or sagittal images should be synchronously quantified for the characteristic, which placed additional complexity on this parameter. PADUA and NePhRO listed endophytic property as one of critical score parameters, but did not provide any detailed methodology for quantifying this characteristic (Ficarra et al., 2009; Hakky et al., 2014). Renal Tumor Invasion Index scoring system stated endophytic property as the maximal diameter of endophytic tumors and appropriate planes of CT images should be chosen depending on tumor location: Axial planes for medial tumors and coronal/sagittal planes for polar tumors (Nisen et al., 2014). In general, different two-dimensional endophytic/exophytic ratios were calculated depending on different methodologies, two of our patients in Fig. 4 demonstrated inconsistency in endophytic/exophytic ratios between 3D images and 2D images.

Longitudinal location also played a vital role in determining complexity of the renal tumor and surgical difficulty. R.E.N.A.L. scoring system scored this component according to tumor location relative to “polar lines”. “Sinus line” was also used in PADUA scoring system. Not only that, recent PISARP scoring system also classified renal tumors into upper, mesorenal or lower pole location, without a detailed description of their reference line (Tannus, Goldman & Andreoni, 2014). Clinicians or radiologists needed to scroll through axial cross-sectional images repeatedly to identify the “polar line” or “sinus line”. The whole process was subjective and abstract, which reduced the concordance rate by approximately 54% as reported by Bylund (Bylund et al., 2012). Based on 3D reconstruction, stereoscopic imaging model allowed clinicians to measure concrete distance between renal tumor and equatorial plane. This concrete distance quantified the longitudinal location more accurately. Variance analysis and covariance analysis by adjusting basic factors revealed close distance from tumor to renal equatorial plane expanded a longer WIT.

Tumor infiltration with UCS or renal sinus may lead to intraoperative damage to such structures. R.E.N.A.L. quantified this characteristic by the distance between tumor and UCS or renal sinus. PADUA described this characteristic as two aspects: relationship with UCS (1 or 2 point) and relationship with renal sinus (1 or 2 point). With extremely high correlation coefficient between UCS and renal sinus reported by Lin Z (Zhou et al., 2015), we combined these two parameters into one: tumors absent a relationship with both UCS or renal sinus were scored 1 point; tumors merely touching UCS or merely touching renal sinus were scored 2 point; tumors synchronously touching UCS and renal sinus were scored 3 point. Urologists were able to quantify this component from multiple viewing angles by rotating 3D models as needed (Figs. 5B, 6B and 7B).

Renal vascular variation was another important parameter included in our 3D scoring system. To achieve a bloodless surgical field, surgeons needed to clamp renal main artery before tumor resection (Klatte et al., 2015). An accurate description of vascular anatomical features played a vital role during tumor excisional process. Traditional 2D images, both in axial, coronal and sagittal planes, could not show the whole distribution of renal vessels. Due to the development of 3D imaging technique, renal vascular anatomical features were accurately presented to us. All contrast CT images in our cohort study were high-qualitatively reconstructed preoperatively (Figs. 5C, 6C and 7C).

3D scoring system correlated most significantly with WIT in spearman correlation analysis, followed by R.E.N.A.L. score (rs = 0.21, *p* = 0.012), centrality index (rs = -0.20, *p* = 0.019) and Preoperative Aspects and Dimensions Used for Anatomy score (rs = 0.20, *p* = 0.019). But we also viewed R.E.N.A.L. score as a valuable nephrometry scoring system because descriptive parameters in R.E.N.A.L., such as “h” for tumor touching main renal artery, could not be calculated in our statistical analysis. Our 3D score presented the most significant correlation with WIT, which reflected the most accurate quantifications for tumor complexity. ROC curve analysis also illustrated that the 3D scoring system could be helpful for clinicians in predicting a long WIT. During the subsequent follow-ups of our cohorts, effects of 3D scoring system and WITs on long-term functional outcomes will also be explored in our next but independent study.

Our present study also had some limitations. First of all, our analysis was carried out on a small sample of patients, whose clinical stages were almost all T1a stages. More patients with tumors of higher stages should be included in the next study. In addition, our cohort study was completely based on laparoscopic PN by an extraperitoneal approach. Patients in an open or robot-assisted approach were also needed to be validated using this 3D score. Furthermore, the analysis during our sample revealed a median WIT of 28 min, with an IQR of 27–30. This range may be too narrow to correctly predict the accuracy of different scoring systems. External validation in multi-centers including a larger range of WIT should also be carried out to prove accuracy of this novel system. Not only that, 3D scoring system depended on high-quality CT images of thin-slice, this new methodology may cost more time. However, considering great accuracy of 3D images and highly developed 3D imaging technique, it deserves our efforts to propose a 3D scoring system to improve clinical experience for PN.

## Conclusions

To sum up, 3D scoring system greatly improved accuracy of measurement for anatomical feature of renal tumors compared to 2D-based scoring systems. The results of our study suggested that 3D scoring system might have great importance in guiding PN planning for clinicians.

##  Supplemental Information

10.7717/peerj.8637/supp-1 Supplemental Information 1 Raw data

## Additional Information and Declarations

### Data Availability

The following information was supplied regarding data availability: The raw data are available in the Supplementary File.
